# Distraction by Novel and Pitch-Deviant Sounds in Children

**DOI:** 10.3389/fpsyg.2016.01949

**Published:** 2016-12-15

**Authors:** Nicole Wetzel, Erich Schröger, Andreas Widmann

**Affiliations:** Cognitive and Biological Psychology, Institute of Psychology, University of LeipzigLeipzig, Germany

**Keywords:** attention, distraction, children, concentration, development, auditory, novelty, oddball

## Abstract

The control of attention is an important part of our executive functions and enables us to focus on relevant information and to ignore irrelevant information. The ability to shield against distraction by task-irrelevant sounds is suggested to mature during school age. The present study investigated the developmental time course of distraction in three groups of children aged 7–10 years. Two different types of distractor sounds that have been frequently used in auditory attention research—novel environmental and pitch-deviant sounds—were presented within an oddball paradigm while children performed a visual categorization task. Reaction time measurements revealed decreasing distractor-related impairment with age. Novel environmental sounds impaired performance in the categorization task more than pitch-deviant sounds. The youngest children showed a pronounced decline of novel-related distraction effects throughout the experimental session. Such a significant decline as a result of practice was not observed in the pitch-deviant condition and not in older children. We observed no correlation between cross-modal distraction effects and performance in standardized tests of concentration and visual distraction. Results of the cross-modal distraction paradigm indicate that separate mechanisms underlying the processing of novel environmental and pitch-deviant sounds develop with different time courses and that these mechanisms develop considerably within a few years in middle childhood.

## Introduction

Attention as a function of cognitive control plays a key role in the acquisition of knowledge about the world. Selective attention can be defined as the allocation of resources to select and enforce the processing of (goal-) relevant stimuli and to inhibit the processing of irrelevant stimuli. Enhanced understanding of the underlying mechanisms and their development is relevant for almost every area of life. Selective attention abilities are relevant in particular for academic achievement (Stevens and Bavelier, 2012). An inspection of the literature on the development of attention reveals that visual attention has been an object of research more frequently than auditory attention (e.g., Rueda et al., 2004). It can be expected that attention processes operate in part differently in the visual and auditory modality as well as cross-modally (Gomes et al., 2000). The present study systematically investigated primary school children's ability to shield against distraction by different types of task-irrelevant sounds while they performed a task. Results of the study enhance our knowledge on the developmental pathway of the control of attention in a cross-modal situation, where auditory distractors are supposed to interfere with a visual primary task.

An example of every-day distraction is the following: In a classroom situation, a child voluntarily focuses attention on the teacher. In doing so, the child shields herself against distraction by task-irrelevant events in order to follow the teacher's presentation. Nevertheless, the child might be distracted by the sound of the bell announcing the end of the lesson. The detection and evaluation of this distractor sound is important for the child since this sound has a high relevance: it announces a period of preferred activity such as playing or talking with friends. When the child is distracted by the new but task-irrelevant sound, performance can be impaired, i.e. the child will miss the information provided by the teacher. This example demonstrates that a permanent balance between voluntary and involuntary aspects of attention control is required.

The mechanisms underlying auditory distraction operate on several stages that have been described by a model that links involuntary and voluntary aspects of auditory attention (e.g., Schröger, 1997; Escera and Corral, 2007; Horvath et al., 2008; Wetzel and Schröger, 2014). It is assumed that when attention is focused to the task-relevant stimulus features, a predictive model of the regular events in the acoustic environment is automatically established on the basis of the current auditory environment (Winkler et al., 2009; Winkler and Schröger, 2015), for a neurophysiologically feasible computational model of detection of potentially important information see also May and Tiitinen (2010). It has been discussed that new and unexpected sounds violate the predictive model (Stage 1) and can cause attentional capture (Stage 2). It has been discussed that orienting the attention on the irregular new or changed sound and then evaluating this sound requires resources which are no longer available for the task at hand. When no further adaption of behavior is required attention is (voluntarily) reoriented back to task-relevant information (Stage 3). The involuntary attention mechanisms in the three stages operate partly independently of each other (Horvath et al., 2008). It is assumed that these mechanisms already function early in life (e.g., Putkinen et al., 2012; Kushnerenko et al., 2013) but develop further (Maurer et al., 2003; Gumenyuk et al., 2004; Wetzel et al., 2006; Mahajan and McArthur, 2015) and with respective different time courses during childhood (Wetzel et al., 2009).

The distraction of attention by new but task-irrelevant events and the underlying mechanisms have been intensively studied in adults (for review see, Schröger and Wolff, 1998; Escera and Corral, 2007). Reliable paradigms for the investigation of sound-related distraction effects are versions of the oddball paradigm. In the auditory oddball or distraction paradigm, new or changed sounds (oddballs) violate a regularity established within a sequence of standard sounds which comprise the regularity while participants perform a visual (Escera et al., 1998) or auditory (Schröger and Wolff, 1998) categorization task. The occurrence of task-irrelevant novel or deviant sounds usually prolongs reaction times or occasionally decreases hit rates in the task at hand. These distraction effects are very stable on the behavioral level and were observed in the auditory, visual, and tactile modality in adults (Bendixen et al., 2010; Ljungberg and Parmentier, 2012). Auditory or cross-modal auditory-visual distraction paradigms have also been successfully performed with children (for review see, Wetzel and Schröger, 2014).

**Figure 1:**
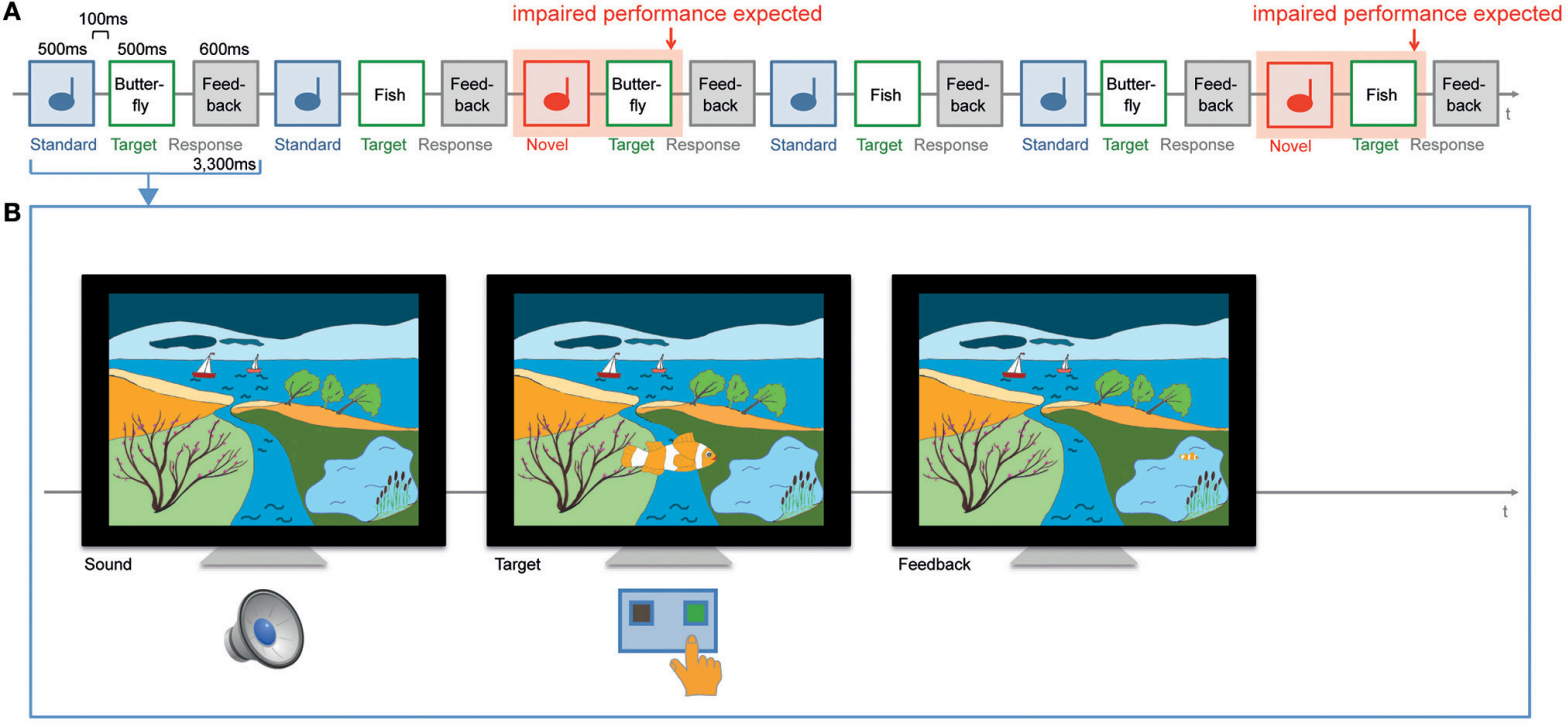
**Displays the distraction paradigm including trial structure. (A)** Rarely and randomly presented novel or pitch-deviant sounds were presented within a sequence of repeated standard sounds (an example of the novel condition is displayed). Sounds were not relevant for the categorization of the following targets (e.g., butterflies and fish). **(B)** Children were asked to distinguish targets by “guiding” target objects to the place where they usually feel comfortable. For example a butterfly would prefer the flowering shrub that is located on the left side and a fish would prefer the pond located on the right side. In the displayed scene the child has to press the right button when a fish appears. After a correct response the fish swims in the pond.

The control of attention is in particular closely related to the maturation of the prefrontal cortex. The prefrontal cortex matures until young adulthood (e.g., Casey et al., 2005). When the control of attention increases with age, it can then be hypothesized that distraction effects decrease with age. A few studies have investigated age effects of oddball sound processing on a task in the visual (Gumenyuk et al., 2001, 2004; Ruhnau et al., 2010, 2013) or auditory modality (Wetzel et al., 2006, 2009; Wetzel and Schröger, 2007b; Horvath et al., 2009; Wetzel, 2015). In the present study, we focused on effects of distractor sounds on performance in the visual modality. In a previous study by Gumenyuk et al. (2001) novel environmental sounds were presented embedded in a sequence of sine wave standard sounds to two groups of children aged 7–10 and 11–13 years while the children performed a visual categorization task. The reaction times of the younger children were more prolonged by task-irrelevant novel sounds than those of the older children. Similar age effects on the distractor-related hit rates have been reported for 8–9-year-old children and 10–11-year-old children during the presentation of a similar cross-modal distraction paradigm (Gumenyuk et al., 2004). The distraction effect did not differ between two groups of older children aged 10–11 and 12–13 years (Gumenyuk et al., 2004). Results indicate that the ability to shield against distraction by novel sounds during a visual task strongly matures during middle childhood and reaches an advanced level of development in late childhood. The assumption of advanced attentional control abilities in late childhood is supported by studies that reported no increased distraction effects in children aged 9–10 years in relation to adults in cross-modal oddball paradigms (Ruhnau et al., 2010, 2013). However, the development of attention control during the age period of 7–10 years has been little investigated in the context of novel processing during a visual task, albeit this is a typical situation in school. Therefore, the first aim of the study was to investigate distraction effects in three groups of children in the age range of 7–10 years. In a cross-modal distraction paradigm, we rarely and randomly presented distractor sounds within a sequence of repeated standard sounds while the children performed a visual categorization task (Figure 1). We measured reaction times and hit rates. We expected decreased distraction effects with increasing age. This hypothesis was based on the maturational time course of the brain, in particular of the prefrontal cortex, during the investigated age range of 7–10 years (Casey et al., 2005), as well as on results of experimental studies about the development of executive functions and inhibitory control in general (Ridderinkhof and van der Molen, 1997) and the absence of developmental effects with slightly older children (e.g., Ruhnau et al., 2010).

The second aim of the study was to investigate the processing of two different types of distractor sounds in middle childhood: novel environmental sounds (termed novels) and pitch-deviant sounds (termed pitch-deviants). Both types of distractor sounds have been frequently used in auditory attention research since they are important for essential aspects of life such as physical integrity (e.g., car horn while crossing a road) or speech comprehension (verbal communication at a party). The difference between natural sounds and sounds that differ only in frequency from standard sounds is important as it can be assumed that the distracting potential is quite different. Novel environmental sounds are physically complex sounds, they have a semantic content, and they are new as they were presented only once per experiment. It has been shown that these features can increase distraction in adults (e.g., Escera et al., 1998; Berti, 2012). It can be assumed that novel environmental sounds have a larger distracting potential and it could be expected that children are more distracted by novel sounds than by pitch-deviant sounds. However, there are an increasing number of studies that discussed the beneficial effects of novel environmental sound processing on performance in children and adults (van Mourik et al., 2007; SanMiguel et al., 2010; Wetzel et al., 2012; Tegelbeckers et al., 2016). It has been discussed that novel sounds could optimize the level of arousal and facilitate processing which results in enhanced performance in novel trials. Therefore, novel environmental sounds might cause less distraction than pitch-deviant sounds in the present study. In order to test these two alternative hypotheses, we presented novel environmental and pitch-deviant sounds within a sequence of standard sounds to the three groups of children. To examine the course of distraction by novel and deviant sounds during the experiment, we additionally analyzed distraction effects as a function of practice. An age-related differential decline of distraction effects with increasing block order enables conclusions regarding the maturational time course of the underlying mechanisms.

Moreover, we compared effects of distraction in our cross-modal paradigm with performance in a paper and pencil concentration test (Intelligence and Development Scales, Grob et al., 2013) as well as with a standardized computer-based visual distraction test (child version of the Test of Attentional Performance, Zimmermann et al., 2002). Although auditory distraction is conceptually related to the concept of selective attention in general and to the concept of visual distraction, there are also differences to these concepts (e.g., Gomes et al., 2000; Wetzel and Schröger, in press). Thus, we were interested in whether and to what extent auditory distraction as measured in the present paradigm correlates with standardized measures of selective attention and visual distraction. We expected that children who have a high performance score in the standardized tests to show improved performance in the present experimental distraction task and reduced distraction effects.

## Methods

### Participants

The study was performed in the after-school center of a primary school in a German city. A total of 50 children participated in the study and 45 of them performed both sessions. Four of them were excluded from analysis because of strong disturbances by incoming people or technical problems. Three children (aged 7, 8, and 9 years) were excluded based on insufficient performance (less than two standard deviations of the mean hit rate in the respective age group). The analysis included 38 children (16 aged 7 years, mean 7 years 5 months, 9 female; 10 children aged 8 years, mean 8 years, 3 months, 6 female; 12 aged 9–10 years, mean 9 years, 8 months, 3 female). Participation was rewarded by age-appropriate gifts and a certificate of participation. Children gave oral and parents gave written informed consent. Parents confirmed their children's normal hearing, normal or corrected-to-normal vision, no medication with effects on the nervous system, and no history of attention-related disorders. The project was approved by the local ethical committee of the Medical Faculty of the University.

### Conditions

We presented two versions of an auditory-visual oddball paradigm (Figure 1). The novel condition included uniquely presented novel environmental distractor sounds. The pitch-deviant condition included two different pitch-deviant distractor sounds. The two conditions were presented in a within-subject design on two separate days in order to keep the experimental time as short as possible. The mean interval between the two sessions was 6 days. Each condition included three blocks with 36 trials each. The order of conditions was balanced between subjects.

### Sounds

The environmental sounds presented in the novel condition were selected from a database described by Wetzel et al. (2011) and have been rated as identifiable sounds (Wetzel et al., 2011). Novels such as bird songs or sneezing were presented only once within a sequence of a repeated environmental standard sound (fragment of a bell). In contrast to some of the previous studies (e.g., Gumenyuk et al., 2001) we did not present novel sounds within a sequence of sine wave standard sounds in order to reduce the influence of differences in bandwidth and sound complexity between standard and distractor sounds. In the pitch-deviant condition, sine wave sounds with frequencies of 500 Hz (77.7%; standard) and 400 (11.1%) and 600 Hz (11.1%) were presented. All sounds had a duration of 500 ms including a raised cosine windowed fade-in and fade-out of 10 ms each. Sounds were presented with a loudness of average 53 dB(A), 55 db SPL. The loudness of sounds was equalized to 6.5 sone (DIN 45631 with diffuse field equalization, Zwicker et al., 1991).

### Task and targets

Participants were asked to distinguish between two different target categories by button press and to ignore the sounds (Figure 1). The target categories differed between the three blocks: (1) princesses and knights (2) cats and chickens (3) butterflies and fish (Figure 1). Within each target category, two versions of the targets were presented slightly differing in shape and color, one oriented toward the left and the other toward the right side. That is, per block four possible targets from two categories (e.g., orange and green fish vs. yellow and purple butterflies) were presented with equal probability (25% each) in pseudo-randomized order. In order to maintain children's motivation and interest, the three blocks included not only different target objects but also a different appropriate background landscape. Princesses and knights were presented in front of a palace and a fortress, cats, and chickens were presented in front of a basket and a chicken roost in a village, and butterflies and fish were presented together with a flowering shrub and a pond embedded in a coastal landscape. Targets and background scenes were the same in the novel and pitch-deviant condition.

The children's task was to press the button that locally matches the preferred and habitual residence of the target object (Figure 1). Children were instructed to press the button as fast and correctly as possible. Correct button presses were rewarded while the target object walked, flew, or jumped to the preferred place. The short presentation of two feedback target stimuli caused the impression of the target objects' movement toward the preferred place. No feedback was given in the case of incorrect or missing responses.

### Standardized tests of concentration and visual distraction

The ability to concentrate was tested using the subtest “Selective Attention” of the Intelligence and Development Scales for children aged 5–10 (Grob et al., 2013). This paper and pencil test consists of several rows of ducks looking to the right or to the left. Children were asked to mark ducks looking to the right which have two orange features (e.g., two orange feet). Children were instructed to mark each row of ducks as quickly and correctly as possible within a certain time period. The raw value includes the number of processed ducks minus the number of wrongly omitted and wrongly marked ducks. The highest possible raw value was 225. Raw values can be transformed into “value points” ranging from 1 to 19 on the basis of age standards (value points 1–6 below-average, 7–13 average, 14–19 above-average).

In order to compare distraction by auditory stimuli with visual distraction, we used the subtest “The enchanted castle” of the standardized computer-based Test of Attentional Performance for Children (KiTAP version 1.5, Zimmermann et al., 2002). Children were asked to press a button if a sad ghost appeared and to not respond if a happy ghost appeared. A variety of visual distractor pictures such as witches or other night creatures were occasionally and randomly presented. Distractor sounds preceded the occurrence of ghosts and were presented within the time range that is usually needed for an eye saccade. The authors stated that if children were distracted by the surrounding non-targets they were not able to distinguish target and non-target ghosts. The test computed reaction times, correct, incorrect and omitted responses as well as age-related *T*-values and percentile ranks in trials with and without distractors.

### Procedure

The children were introduced by the teacher to the experimenter. The experimenter explained the “game.” Each of the three blocks was preceded by a training session in order to familiarize participants with the block-specific targets and backgrounds. The training was not limited in time and included two distractor sounds and six standard sounds. Novel sounds that had been presented in the training blocks were not presented in the experimental blocks. The training block was repeated in case more than 50% of trials were not correctly responded (only one child needed a repetition). The order of blocks was balanced. Distractor sounds were pseudo-randomly presented with a probability of 22.2% (8 per block). Each distractor sound was preceded by at least two standard sounds in order to enable the generation of an auditory regularity of standard sounds. The allocation of sound types to the following target category was balanced. The experimental trial duration was 3300 ms (Figure 1), block duration was about 2 min and the duration of the complete distraction paradigm was about 6 min without breaks. The standardized tests of concentration and visual attention were performed after the experimental task. Per session one of the two tests was performed. The order of tests was balanced. The distraction paradigm was presented using a MacBook (13.3″) via Matlab (The MathWorks, U.S.A.) and the Psychtoolbox (Kleiner et al., 2007). Sounds were presented via external loudspeakers (Bose Corporation, U.S.A) placed on the left and right of the notebook display. To collect responses, two large external response buttons placed on the left and the right side of the notebook were used. The response buttons were connected with an RTBox (Lee et al., 2012), wich provided accurate response time measurements.

### Data analysis

The first and the second standard trial in a block as well as the first standard trial after a novel trial were removed from the analysis. A mixed-model analysis of variance (ANOVA) including the within-subject factors *sound type* (standard, distractor), *condition* (novel, pitch-deviant), and the between-subject factor *age* (7 years, 8 years, 9–10 years) was used to analyze mean reaction times, hit rates and omission error rates. In order to test the processing of novel and pitch-deviant sounds throughout the experiment, distractor-minus-standard difference values (distraction effects) were tested using an ANOVA with the factors *condition* (2), *block order* (first vs. second vs. third presented block), and *age* (3). To test relations between sound processing (reaction times, distraction effects) and the ability to concentrate (raw values), we performed a Pearson's correlation separately for conditions. To account for the additional influence of the factor age, a partial correlation was performed. We compared visual distraction effects measured using the standardized KiTap attention test with distraction effects measured using the experimental oddball paradigm. The partial correlation included the mean reaction time difference between trials with and without distractors (KiTap), the mean reaction time difference between distractor and standard trial (experimental oddball paradigm), and age. Our analysis is based on KiTap data that were corrected after the experiment by the editor of the KiTap since delayed response times were incorrectly classified in the original data. A probability *p*-value of less than 0.05 was considered as significant. Greenhouse-Geisser corrections were applied when appropriate. Follow-up *t*-tests were run for statistically significant interactions and were Bonferroni-corrected if necessary. All statistical analyses described above were performed using Matlab (The MathWorks, U.S.A) and SPSS (IBM, U.S.A.).

## Results

### Reaction times, hit rates, and omission rates

**Figure 2:**
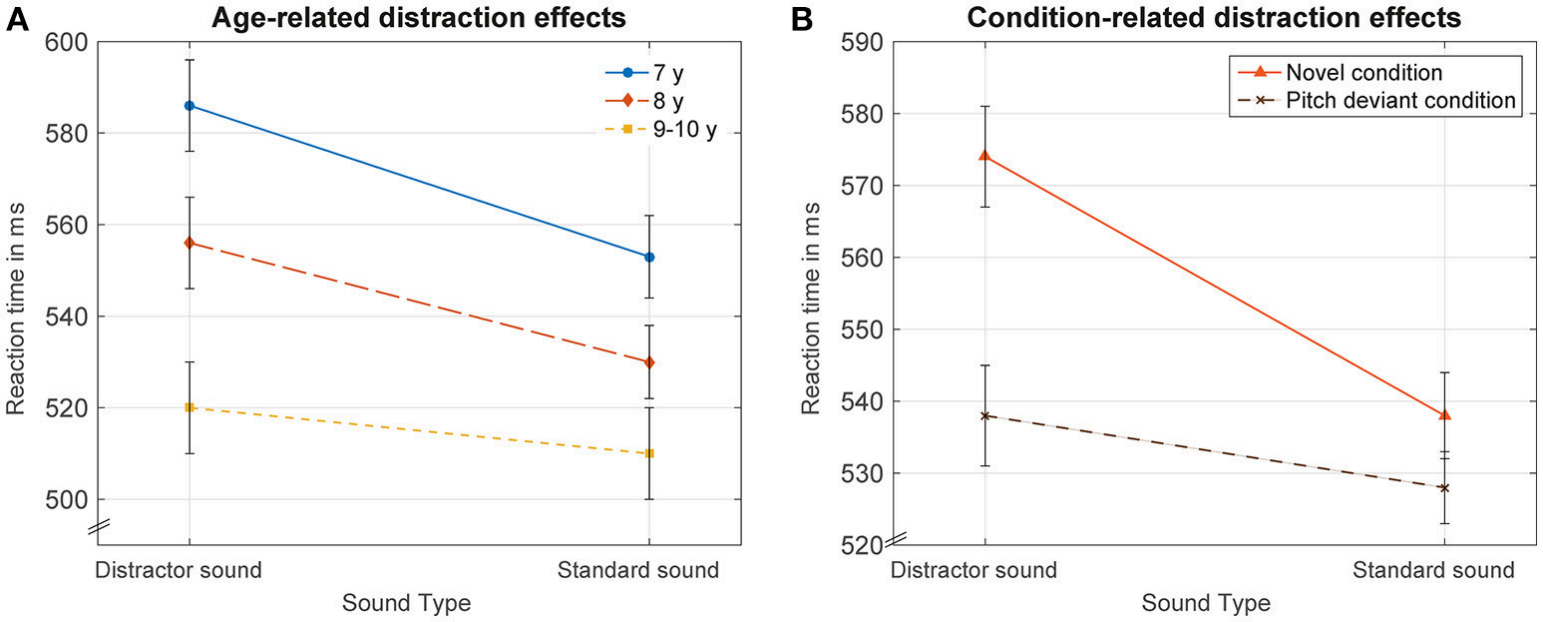
**Displays age and condition effects resulting from the statistically significant interactions of the factors *sound type* × *age* and *sound type* × *condition***. **(A)** Reaction times (mean over conditions) in distractor and standard trials are displayed for each age group. Reaction times were increased in distractor compared to standard trials in all age groups. This distraction effect decreased significantly with age. **(B)** Reaction times (mean over age) in distractor and standard trials for each condition. Novel environmental sounds caused increased reaction times compared to pitch-deviant sounds. Reaction times in response to standard sounds did not differ significantly between conditions. Error bars reflect the standard error of mean.

The ANOVA with the factors *sound type (2)* × *condition (2)* × *age (3)* revealed that distractor sounds prolonged reaction times in comparison to standard sounds [main effect sound type: *F*_(1, 35)_ = 44.34, *p* ≤ 0.001, η_*p*_^2^ = 0.56]. Reaction times in the novel condition were increased compared to reaction times in the pitch-deviant condition [main effect condition: *F*_(1, 35)_ = 6.65, *p* ≤ 0.014, η_*p*_^2^ = 0.16]. Distraction effects differed between groups [interaction of the factors *sound type* × *age*: *F*_(2, 35)_ = 3.82, *p* ≤ 0.031, η_*p*_^2^ = 0.18; see Figure 2]. Distractor-related impairments decreased with age [*t*-tests distractor vs. standard; Bonferroni correction applied: 7-year-old: *t*_(15)_ = 5.42, *p* ≤ 0.001, 33 ms; 8-year-old: *t*_(15)_ = 3.30, *p* ≤ 0.009, 26 ms; 9–10-year-old: *t*_(11)_ = 3.65, *p* ≤ 0.004, 11 ms]. Importantly, novel sounds elicited prolonged reaction times compared to pitch-deviant sounds, whereas reaction times in standard trials did not differ between the conditions [interaction of the factors *sound type* × *condition*: *F*_(1, 35)_ = 8.06, *p* ≤ 0.007, η_*p*_^2^ = 0.19; *t*-test novel vs. pitch-deviant: *t*_(37)_ = 3.21, *p* ≤ 0.003; *t*-test standards in the novel condition vs. standards in the pitch-deviant condition: *t*_(37)_ = 1.06, *p* = 0.296]. The interaction including the factors *condition* × *age* was not statistically significant [*F*_(2, 35)_ = 1.38, *p* = 0.265, η_*p*_^2^ = 0.07]. The interaction including the factors *sound type* × *condition* × *age* was not statistically significant (*F* < 1). The main effect of age was not statistically significant [*F*_(2, 35)_ = 2.22, *p* = 0.123, η_*p*_^2^ = 0.11]. When including the factor *gender* (female, male) in the analysis, no gender-related effects were observed.

The hit rate ranged from 89 to 96% (Table 1). No statistically significant effects were found for the hit rate. Results revealed *F*-values < 1 except for the main effect of age [*F*_(2, 35)_ = 1.17, *p* = 0.322, η_*p*_^2^ = 0.06] and the three-way interaction [*F*_(2, 35)_ = 1.47, *p* = 0.244, η_*p*_^2^ = 0.08]. The omission rate was larger in the oldest group than in both younger groups [main effect age: *F*_(2, 35)_ = 5.824, *p* ≤ 0.007, η_*p*_^2^ = 0.25, Scheffé-test: 7 years vs. 9–10 years: *p* ≤ 0.029, 8 years vs. 9–10 years: *p* ≤ 0.014]. No further interactions or main effects were statistically significant (all *F*-values < 1.3).

**Table 1:** **Mean reaction times (RT), hit and omission rate in % and standard deviation (SD)**.

**Age group**	**Novel condition**			**Pitch-deviant condition**		
	**Distraction effect**	**Novel**	**Standard**	**Distraction effect**	**Deviant**	**Standard**
**RT (SD) IN MS**						
7 years	^***^51.2 (50.0)	606.9 (92.3)	555.7 (88.6)	14.4 (34.7)	564.3 (83.6)	549.9 (68.5)
8 years	^*^35.8 (44.2)	582.8 (88.3)	547.0 (72.6)	^*^15.2 (19.3)	528.3 (49.9)	513.1 (43.5)
9–10 years	^***^20.8 (15.0)	527.3 (70.3)	506.5 (72.6)	0.8 (20.5)	513.3 (68.1)	512.4 (70.3)
**HIT RATE (SD) IN %**						
7 years	1.0 (4.9)	92.4 (7.9)	91.4 (7.0)	2.0 (4.6)	91.5 (8.1)	89.5 (7.6)
8 years	−0.3 (5.1)	92.2 (7.7)	92.4 (4.0)	0.1 (5.7)	93.0 (6.0)	92.9 (3.4)
9–10 years	2.0 (6.1)	94.8 (6.3)	92.8 (5.3)	−1.2 (6.6)	94.2 (6.5)	95.5 (4.9)
**OMISSION RATE (SD) IN %**						
7 years	0.1 (1.7)	0.8 (1.7)	0.7 (0.9)	0,0 (0.5)	0.5 (1.4)	0.5 (1.2)
8 years	0.5 (2.1)	0.8 (1.8)	0.3 (0.8)	−0.4 (0.8)	0.0 (0.0)	0.4 (0.8)
9–10 years	0.9 (3.5)	2.1 (2.8)	1.2 (1.8)	0.2 (2.2)	1.7 (2.1)	1.5 (2.0)

*Distraction effects = distractor (novel or pitch-deviant) minus standard*.

Asterisks mark statistically significant differences between distractor and standard reaction times (t-test comparison):

*** p ≤ 0.001,

* *p ≤ 0.05*.

To control for possible effects of target category (or background scene) and deviant pitch (high or low; pitch-deviant condition only) we computed separate ANOVAs on the distraction effect. Neither target category (main effect and interactions including target category *F* < 1) nor deviant pitch [RT: deviant type × group *F*_(2, 35)_ = 1.18, *p* = 0.319, η_*p*_^2^ = 0.063; main effect deviant type *F*_(1, 35)_ = 0.06, *p* = 0.802, η_*p*_^2^ = 0.002; hit rate: deviant type × group *F*_(2, 35)_ = 1.47, *p* = 0.244, η_*p*_^2^ = 0.077, main effect deviant type *F*_(1, 35)_ = 1.43, *p* = 0.239, η_*p*_^2^ = 0.039] significantly modulated the distraction effect.

### Distraction effects as a function of block order

**Figure 3:**
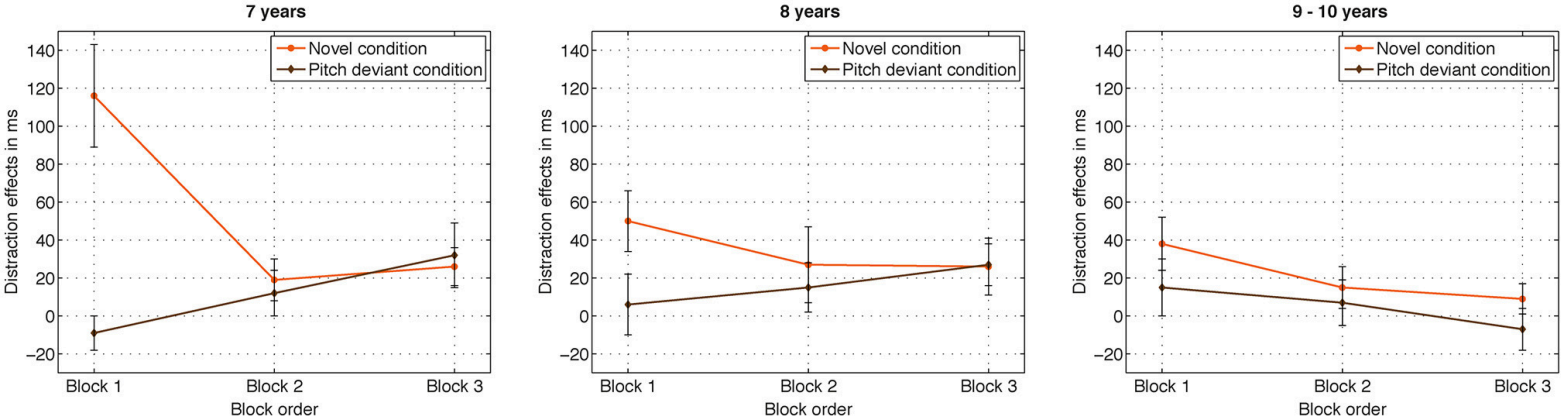
**Highlights the different course of distraction effects (distractor RT minus standard RT) throughout the session in the different age groups**. Distraction effects significantly decreased from the first to the second block in the novel condition but not in the pitch-deviant condition in the 7-year-old children. A similar but less pronounced pattern was observed in the 8-year-old children but not in the 9–10-year-old children.

In order to test the course of distraction as a function of practice, we compared RT distraction effects (distractor minus standard) in the novel and pitch-deviant conditions between blocks. The distraction effects of the youngest children significantly decreased from the first to the second presented block in the novel condition (Figure 3). This strong decrease was not observed for the pitch-deviant condition and not for the older children. The interaction of the factors *condition* × *block order* × *age* was statistically significant [*F*_(4, 70)_ = 3.94, *p* < 0.009, η_*p*_^2^ = 0.18, ε = 0.885]. The follow-up interaction of the factors *condition* × *block order* was statistically significant only in the youngest children [*F*_(2, 30)_ = 17.25, *p* ≤ 0.001, η_*p*_^2^ = 0.54, ε = 0.793; middle children *F*_(2, 18)_ = 1.41, *p* = 0.269, η_*p*_^2^ = 0.14, ε = 0.965; older children *F* < 1]. The follow-up *t*-test for the youngest children revealed larger distraction effects in the novel condition in the first block than in the second block [*t*_(15)_ = 3.87, *p* ≤ .002] whereas distraction effects did not differ between the second and the third presented block [*t*_(15)_ = −0.451, *p* = 0.659]. In contrast to the novel condition, distraction effects numerically increased throughout the experiment in the youngest children in the pitch-deviant condition, but these order effects missed statistical significance (first block vs. third block: *t*_(15)_ = −1.965, *p* = 0.068; first block vs. second block: *t*_(15)_ = −1.832, *p* = 0.087; second vs. third block: *t*_(15)_ = −0.953, *p* = 0.356).

### Correlation of distraction effects with performance in the standardized tests

The number of mean value points in the IDS concentration test was 10. Twenty-eight (74%) children performed the IDS concentration test at average level (7–13 value points). Seven (18%) children performed at below-average level (4–6 value points). Three (8%) children performed at above-average level (14–19 value points). Children who performed better in the IDS concentration test (raw values) responded faster in distractor (*r* = −0.611, *p* ≤ 0.001) and standard trials (*r* = −0.568, *p* ≤ 0.001) in the pitch-deviant condition. The pattern of this relation was similar but less pronounced in the novel condition (novel: *r* = −0.315, *p* = 0.058; standard: (*r* = −0.381, *p* ≤ 0.020). No relation between distraction effects (distractor minus standard) and performance in the concentration test (raw values) was observed (novel condition: *r* = 0.087, *p* = 0.608; pitch-deviant condition: *r* = −0.255, *p* = 0.128). No statistical significant correlation between hit rate (all *r* < 0.213) or omission rate (all *r* < 0.145) and performance in the concentration test was observed.

Commission and omission error rate and median RT in the standardized visual distraction test of the KiTap revealed the following results (mean *T* = 50, one standard deviation = 10). Commission errors: 17 (45%) children scored *T* < 40, 19 (50%) children scored *T*-values between 40 and 60, and two (5%) children scored *T* > 60. Omission errors: eight (21%) children scored *T* < 40, 17 (45%) children scored *T*-values between 40 and 60, 13 (34%) children scored *T* > 60, median RT: 21 (55%) children scored *T*-values between 40 and 60, 17 (45%) children scored *T* > 60. The analysis of the KiTap mean RT, commission and omission errors using an ANOVA with the factors *distractors (trials including visual distractors vs. trials without visual distractors)* and *age* (3) revealed no statistically significant interactions (all *F* < 1.1). Reaction times were 26 ms faster in go-trials including visual distractors compared with go-trials including no distractors, but this result revealed no statistical significance [481 ms vs. 507; *F*_(1, 35)_ = 3.32, *p* = 0.077, η_*p*_^2^ = 0.087]. The number of commission errors showed a tendency to increase in trials including distractors [10 vs. 9; *F*_(1, 35)_ = 3.27, *p* = 0.079, η_*p*_^2^ = 0.085]. No age effects were observed (all *F* < 1.5). Visual distraction effects measured by mean reaction times and commission errors in trials with and without distractors (KiTap) did not correlate with distraction effects in the experimental oddball paradigm (novel condition: *r* = −0.199, *p* = 0.298; pitch-deviant condition: *r* = 0.176, *p* = 0.296).

## Discussion

We aimed to systematically disentangle effects of task-irrelevant novel environmental and pitch-deviant sounds on performance in a visual task in three groups of children aged 7, 8, and 9–10 years. These sound types of distractor sounds have been frequently used in auditory attention research. The main results revealed (1) decreasing distraction effects with increasing age, (2) different age effects on the process of distraction as a function of practice between novels and pitch-deviants, (3) stronger distraction effects elicited by novel sounds than by pitch-deviant sounds and (4) no relation between distraction effects in the experimental task and performance in the standardized concentration and KiTap distraction test.

### Age effects on distraction by novel and pitch-deviant sounds

Distraction effects have been observed in all age groups in the present auditory-visual oddball task. This means that reaction times were prolonged when visual targets were preceded by a distractor sound in relation to targets that were preceded by a standard sound. In fact, 37 out of 38 children showed a behavioral distraction effect in response to novel or pitch-deviant sounds. Importantly, the effects of task-irrelevant sound processing on performance decreased with age, i.e., the distractor-related performance was more impaired in younger than in older children. Results confirm our hypothesis and indicate a significant development of attentional control during the age of 7–10 years.

Most important, the analysis of distraction effects as a function of practice throughout the experiment revealed considerable age differences. In the novel condition, we observed distraction effects of 116 ms in the first presented block in the youngest children (Figure 3). This distraction effect significantly declined after the first block (by 97 ms). Distraction effects did not significantly differ between the second and the third block (19 vs. 26 ms). Both groups of older children also showed a numerical a decline of distraction effects from the first to the second block in the novel condition, but this decrease was considerably smaller (by 23 ms, Figure 3). In the literature, slight decreases of effects of novelty from the first to the forth block have been also observed in adults when presenting an auditory oddball stimulation including sounds and words while participants performed a visual task (Parmentier, 2008). On the basis of our results, we hypothesize that age effects on distraction by novel sounds are composed of several factors. One factor is the basic level of distraction that is assumed to differ between age groups independent of the block order. Distraction effects in the final block decrease with age, but it cannot be excluded that distraction effects would have declined further if more blocks had been presented. A future study including more blocks could investigate when exactly distraction effects reach a plateau in several age groups. The magnitude of the distraction effect in the second and third presented blocks indicates that the proportion of basic age differences might be smaller than assumed previously. In contrast, experience seems to play a critical role for the magnitude of distraction in the different age groups. It can be hypothesized that younger children initially evaluate a number of task-irrelevant novel sounds in detail causing the increased distraction effects in the first block. In contrast, older children were able to control distraction by novel sounds more successfully at an early stage of the experiment. They potentially are able to categorize novel sounds as task-irrelevant and prevent detailed evaluation despite the novelty of sounds. Another explanation could be that pitch-deviant sounds were repeated during the experiment causing habituation of the orienting of attention. However, since novels were never repeated during the experiment, the repetition hypothesis cannot completely explain the abrupt decline of distraction effects in the novel condition. Furthermore, the results of a similar auditory-visual oddball study by Berti (2012) do not support this hypothesis. Berti observed comparable distraction effects in adults in response to uniquely and repeatedly presented environmental oddball sounds. The repetition hypothesis can also not explain the slight increase of distraction effects as a function of practice in both groups of younger children (Figure 3). Our results support the assumption of different developmental time courses of control of attention in the context of novel environmental and pitch-deviant sounds that should be considered in future research.

The age effects on behavioral distraction reported in the present study are in line with research on the development of the underlying brain mechanisms. The occurrence of a sound that differs from the predicted model that has been built up on the basis of the regularities in the acoustic environment, can cause attention to be oriented toward the new sound and its evaluation. Orienting and distractor evaluation mechanisms have been associated with the event-related potential (ERP) P3a or noveltyP3 in the EEG (for review see, Escera et al., 2000; Friedman et al., 2001; Polich, 2007). ERP-studies with children that focused on the ability to control involuntary orienting mechanisms reflected by the P3a reported immature control. When children aged 6–8, 10–12 and adults were asked to attend sound duration within an oddball paradigm, unexpected and task-irrelevant changes in pitch caused orienting of attention and deviant evaluation in all age groups (reflected by P3a, Wetzel et al., 2006). When participants were asked to ignore the same sound sequence and to focus attention on a video clip, then children, in particular the youngest children, nevertheless showed a P3a component, indicating involuntary orienting of attention toward pitch changes. In contrast, adults did not show a P3a when instructed to ignore sounds, indicating successful control of attention orienting. Similar results were observed in an oddball study presenting environmental distractor sounds to 9–10-year-old children (Wetzel, 2015). Also 13-year-old children were more impaired by distractor stimuli in a visual-spatial working memory task than adults (Olesen et al., 2007). The fMRI study reported that children showed stronger activity in the superior frontal sulcus in response to distractor stimuli, which was not observed in adults. The authors discuss that this may explain children's increased susceptibility to distractor stimuli (Olesen et al., 2007).

We observed no effects of sound type, condition, or age on the hit rate or the omission rate. The mean hit rate was 93% indicating that children can easily cope with the task. In the literature, one study reported age effects on the distractor-related hit rate (Gumenyuk et al., 2004), while others did not (Gumenyuk et al., 2001; Ruhnau et al., 2010, 2013). The level of task performance might affect novel related effects on the hit rate. It cannot be excluded that distractor-related impairments of accuracy might be observed if the task were more difficult. However, since we focused on reaction time effects, we decided to present an easy task in order to maintain children's motivation across the two sessions.

In summary, our results specify the developmental time course of distraction by unexpected and task-irrelevant sounds during the age of 7–10 years. The ability to shield against distraction by unexpected and task-irrelevant sounds undergoes significant maturation in middle childhood and increases with age.

### Age-independent effects of novel environmental and pitch-deviant sounds on distraction

Reaction times differed in distractor but not in standard trials between the conditions. Novel environmental sounds elicited larger reaction time prolongation than pitch-deviant sounds (Figure 2). The increased distraction potential of novel environmental compared with pitch-deviant sounds is in line with ERP-studies comparing the effects of these sounds on P3a or noveltyP3. In an auditory-visual oddball study with participants aged 6–18 years, novel environmental sounds elicited increased orienting and distractor evaluation mechanisms reflected by increased P3a amplitudes compared with pitch-deviant sounds (Wetzel and Schröger, 2007a). This result has been replicated in a group of 5–12-years-old children by (Brinkman and Stauder, 2008). Similar pattern of results was observed in adults (e.g., Alho et al., 1998; Gaeta et al., 2003; Berti, 2012). Results of the present study confirm our hypothesis that postulated that novel sounds have a higher distraction potential resulting in increased impairments in a task at hand. The higher distraction potential can be caused by several features differing between novel and pitch-deviant sounds such as complexity, meaning, and novelty. Environmental sounds have a complex structure since they contain a variety of frequencies, whereas pitch-deviant sounds contain only a single frequency. Even infants processed physically complex oddball sounds (e.g., bursts of noise) differently from pitch-deviant sounds (Wetzel et al., 2016). In their auditory-visual oddball study, the distractor-related pupil dilation response was clearly increased in response to noise oddball sounds (that included a variety of frequencies), whereas no response was observed in response to pitch-deviant sine wave sounds (included a single frequency). Furthermore, in contrast to the pitch-deviants, novel environmental sounds provided a meaning that can affect control of attention. It has been reported that the sound of the one's own smartphone captures more attention than the sounds of other smartphones when presented in an oddball paradigm to adults (Roye et al., 2007). Even infants responded to oddball sounds, that provided an emotional meaning (e.g., the cry of a peer), with increased pupil dilation than in response to a phone ring which had a similar complexity but a probably less significant meaning (Wetzel et al., 2016). Semantic information therefore might increase the personal significance of novel sounds contributing to differences between the conditions. A further important difference is the novelty of the distractor sounds. Novel sounds were presented only once and were indeed new in the experimental setting. In contrast, the two pitch-deviants were repeated several times. It has been reported in adults that the processing of novel and pitch-deviant sound activates partly different neural networks (e.g., Escera et al., 1998). The authors suggested that different brain mechanisms involved in change detection were triggered by the different types of sounds. There is some evidence for the auditory modality indicating that even newborns differently process deviancy and contextual novelty and that this separation continues to develop beyond the first year of life (Kushnerenko et al., 2013). It can be assumed that this development also continues into the school-age years since pitch-deviant sounds as well as novel sounds caused prolonged reaction times in children in the present study, whereas in a similar adult study, only novel sounds caused reaction time prolongations (pitch-deviants caused reduced hit rates in adults, Escera et al., 1998, for similar RT effects see also Berti, 2012). The assumption of an ongoing development of the underlying processes is supported by the opposite course of novel- and pitch-deviant-related distraction effects throughout the session in the 7-year-olds.

Our alternative hypothesis of decreased distraction effects in response to novel sounds was not confirmed by the data. This hypothesis was based on adult studies that reported a facilitated processing of targets preceded by novel compared with repeated deviant sounds (SanMiguel et al., 2010; Wetzel et al., 2012). It has been discussed that distraction effects include both, the costs of the orienting of attention away from task-relevant features as well as the benefits of an increase of arousal in response to novel sounds (Näätänen, 1992). The distraction effects could be affected by attentional focusing induced by the task and temporal links between the distractor and target (SanMiguel et al., 2010). In a study focusing on involuntary attention in typically developed children and children suffering from ADHD (8–13 years), novel sounds improved performance in an Eriksen flanker task in both groups of children (Tegelbeckers et al., 2016). The authors did not present an oddball design, but rather presented equiprobably task-irrelevant repeated standard sounds, novel sounds, or silence. The hit rate of the typically developed children was rather similar to those of our study indicating a similar level of task-difficulty. It can be assumed that either the features of the task or the presented frequency of the novel sounds modulate performance. This assumption is supported by findings of a study that investigated typically developed children and children with ADHD (8–12 years) and used a similar design as in the present novel condition (van Mourik et al., 2007). The authors reported prolonged reaction times but reduced error rates in response to novel sounds. Children with ADHD significantly reduced errors of omission after the presentation of a novel sound. This has been discussed in the context of an increase of arousal to an optimal level (van Mourik et al., 2007). However, under the present experimental conditions, no facilitated processing of novel sounds compared with pitch-deviant sounds were observed in children, indicating that the costs of orienting outbalanced any potential benefits of facilitated novel processing.

In summary, larger distraction effects in response to novel environmental sounds than to pitch-deviant sound have been observed in 7–10-year-old children. Future studies are required in order to disentangle which of the sound features discussed above or which combination of sound features contribute to the different distraction effects.

### Relation between cross-modal distraction effects and the ability to concentrate or to shield against visual distraction

The ability to concentrate inversely correlates with reaction times in standard and novel trials. This indicates that children with an increased ability to concentrate responded faster to the visual targets or vice-versa. The less pronounced relation between the speed of responses in novel trials (relative to pitch-deviant trials) and the ability to concentrate might be caused by a broader variability of response times in the novel condition. This supports the assumption of partly different processing of novel and pitch-deviant sounds. The authors of the IDS concentration test depicted that the concentration test measures basic cognitive functions such as to memorizing target features, focusing attention on target features, inhibiting of distractor features, sustaining attention as well as the flexibility to apply several rules. Similar cognitive abilities were required in order to successfully perform the experimental distraction task. Therefore, we expected that performances in the concentration test and experimental distraction task would correlate with each other to a certain degree. However, our statistical analysis revealed no relation between distraction effects and the ability to concentrate. On the one hand, this could indicate that the mechanisms underlying the processing of distractor sounds are not directly related to the ability to concentrate. One reason could be that the concentration test requires cognitive functions that exclusively gain input from the visual modality. In contrast, the experimental distraction task requires cross-modally operating mechanisms that might require cognitive abilities that are not reflected in the performance on the concentration test. If this assumption is true, then additional cross-modal concentrations tests should be used to measure selective attention abilities in children. Since distraction in every-day life often occurs in cross-modal situations, this would be a more ecologically valid measurement. On the other hand, it is possible that children performed too homogeneously in the standardized concentration test. Our study focused on healthy children. Children with attention problems, that often show impaired performance in concentration tests, did not participate in this study.

In the standardized visual distraction test (KiTap), children tended to respond faster and less correctly to targets in the condition that included visual distractor stimuli. Results could be caused by an accuracy-speed trade off. A study that tested the construct validity of this subtest of the KiTap in a similar age range reported a reverse pattern: increased reaction times and reduced commission errors in the distractor condition (Schöneck et al., 2014). However, the authors also discussed that the results might have been caused by an accuracy-speed trade off. We observed no relation between the distractor-related performance in the visual distraction test and the distraction effects elicited by the auditory distractors in the experimental task. This result supports the statement by Gomes et al. (2000) who emphasized that the (advanced) knowledge on the development of visual attention cannot be simply assigned to the research on the development of auditory attention. The present study revealed distinct distraction effects that were sensitive to the type of the eliciting sound and that differed between age groups—emphasizing the need of developmental attention research in the auditory modality. The integration of both fields of attention research can contribute to the development of general models of attention and can enhance knowledge of the developmental time course of attention. Moreover, the present paradigm was not only sensitive to attentional control abilities but was also suitable for children since it was less time consuming, playful and most of the children enjoyed participating. Therefore, similar paradigms might be useful in order to investigate typically developed children including preschool children as well as atypically developed children including children suffering from attention disorders.

## Conclusion

The effects of the ongoing maturation of involuntary attention mechanisms were observed on the behavioral level in children aged 7–10 years. The performance in a concomitant visual task was more impaired by task-irrelevant unexpected sounds in younger than in older children. Novel environmental sounds compared with sounds that differ in pitch from the auditory context appear to have a higher distracting potential for children because they cause increased distraction effects. The magnitude of distraction effects throughout the experimental session differed between the age groups as a function of practice. In particular, the youngest children were highly distracted by novel environmental sounds but not by pitch-deviant sounds at the beginning of the auditory stimulation. We conclude that the underlying attention mechanisms develop considerably between the age of 7 and 9/10 years. Results of the present study emphasize the importance of a specific age-related adaption of learning environments to the needs of children. Moreover, our results strengthen the trend in auditory developmental research to present everyday environmental sounds in order to enhance the ecological validity.

## Ethics statement

All children gave oral and all parents gave written informed consent. All parents gave written informed consent in accordance with the Declaration of Helsinki. The protocol was approved by the Ethic Commission of the Medical Faculty of the University of Leipzig.

## Author contributions

All authors listed, have made substantial, direct and intellectual contribution to the work, and approved it for publication.

## Funding

This project was supported by the German Research Foundation (DFG) project number WE5026/1-2 and by the University of Leipzig.

### Conflict of interest statement

The authors declare that the research was conducted in the absence of any commercial or financial relationships that could be construed as a potential conflict of interest.
